# Integrative Analysis of Transcriptomic Profiles and Physiological Responses Provide New Insights into Drought Stress Tolerance in Oil Palm (*Elaeis guineensis* Jacq.)

**DOI:** 10.3390/ijms25168761

**Published:** 2024-08-12

**Authors:** Fernan Santiago Mejía-Alvarado, Arley Fernando Caicedo-Zambrano, David Botero-Rozo, Leonardo Araque, Cristihian Jarri Bayona-Rodríguez, Seyed Mehdi Jazayeri, Carmenza Montoya, Iván Ayala-Díaz, Rodrigo Ruiz-Romero, Hernán Mauricio Romero

**Affiliations:** 1 Biology and Breeding Research Program, Colombian Palm Oil Research Center, Cenipalma, Calle 98 No. 70-91, Piso 14, Bogotá 111121, Colombia; fmejia@cenipalma.org (F.S.M.-A.); acaicedo@cenipalma.org (A.F.C.-Z.); dbotero@cenipalma.org (D.B.-R.); laraque@cenipalma.org (L.A.); cbayona@cenipalma.org (C.J.B.-R.); smjazayeri@unal.edu.co (S.M.J.); cmontoya@cenipalma.org (C.M.); iayala@cenipalma.org (I.A.-D.); rruiz@cenipalma.org (R.R.-R.); 2 Department of Biology, Universidad Nacional de Colombia, Bogotá 111321, Colombia

**Keywords:** water stress, water use efficiency, RNA-Seq, gene coexpression networks, phytohormone crosstalk, signal cascade

## Abstract

Oil palm (*Elaeis guineensis* Jacq.) is a highly productive crop economically significant for food, cosmetics, and biofuels. Abiotic stresses such as low water availability, salt accumulation, and high temperatures severely impact oil palm growth, physiology, and yield by restricting water flux among soil, plants, and the environment. While drought stress’s physiological and biochemical effects on oil palm have been extensively studied, the molecular mechanisms underlying drought stress tolerance remain unclear. Under water deficit conditions, this study investigates two commercial *E. guineensis* cultivars, IRHO 7001 and IRHO 2501. Water deficit adversely affected the physiology of both cultivars, with IRHO 2501 being more severely impacted. After several days of water deficit, there was a 40% reduction in photosynthetic rate (*A*) for IRHO 7001 and a 58% decrease in IRHO 2501. Further into the drought conditions, there was a 75% reduction in *A* for IRHO 7001 and a 91% drop in IRHO 2501. Both cultivars reacted to the drought stress conditions by closing stomata and reducing the transpiration rate. Despite these differences, no significant variations were observed between the cultivars in stomatal conductance, transpiration, or instantaneous leaf-level water use efficiency. This indicates that IRHO 7001 is more tolerant to drought stress than IRHO 2501. A differential gene expression and network analysis was conducted to elucidate the differential responses of the cultivars. The DESeq2 algorithm identified 502 differentially expressed genes (DEGs). The gene coexpression network for IRHO 7001 comprised 274 DEGs and 46 predicted HUB genes, whereas IRHO 2501’s network included 249 DEGs and 3 HUB genes. RT-qPCR validation of 15 DEGs confirmed the RNA-Seq data. The transcriptomic profiles and gene coexpression network analysis revealed a set of DEGs and HUB genes associated with regulatory and transcriptional functions. Notably, the zinc finger protein *ZAT11* and linoleate 13S-lipoxygenase 2-1 (*LOX2.1*) were overexpressed in IRHO 2501 but under-expressed in IRHO 7001. Additionally, phytohormone crosstalk was identified as a central component in the response and adaptation of oil palm to drought stress.

## 1. Introduction

Low water availability, salt accumulation on land, and high temperatures are the main abiotic conditions that restrict the water flux among soil, plants, and the environment. Water deficit eventually results in drought stress, affecting plants’ morphological, physiological, biochemical, and genetic conditions [1]. Drought stress has been well studied in several commercial crops, and the most common symptoms are growth reduction and yield loss [2]. To overcome stressful conditions, plants trigger several mechanisms, overlapping some processes in different plant tissues [3]. Under limited conditions, plant responses will occur according to the occurrence and intensity of stress events; in this sense, these responses might activate diverse strategies for avoidance, tolerance, or escape of stressor conditions [4]. Under early or moderate drought conditions, plants avoid losing water by closing their stomata and using water efficiently. Moreover, plants adjust solute concentrations to tolerate temporal or severe drought stress conditions, decrease photosynthesis, and encode desiccation-tolerant proteins. Finally, plants accelerate their vegetative cycle, leading to early maturation and escape from prolonged or critical drought stress conditions [5].

Oil palm (*Elaeis guineensis* Jacq.) is an important economic crop for foods, cosmetics, and biofuels because its high yield per hectare is higher than other oilseed crops such as soybeans, sunflower, and canola [6]. *E. guineensis* is a monocotyledon plant of the *Arecaceae* family that originated in tropical areas of Africa and expanded to humid regions of Malaysia, Indonesia, and the Americas [7]. In oil palm, water availability is one of the main limiting factors in the vegetative and reproductive stages. A direct association has been found between water volume requirements (mm water day^−1^) and yield (tons fruit bunches ha^−1^ year^−1^) [8,9]. To maintain high yield levels, oil palm crops require 1200 to 4000 mm year^−1^ of rainfall; this range might change according to the growing area and cultivar [9,10]. In many countries, oil palm plantations are exposed to extended dry seasons, one of the main limiting factors of climatic pattern fluctuations [6].

The physiological and biochemical effects of drought stress in oil palm have been well studied in *E. guineensis* [11,12] and the interspecific hybrid O×G (*Elaeis oleifera* (Kunth) Cortés × *Elaeis guineensis* Jacq.) [13], suggesting drought stress cultivar-dependent tolerance [11,14]. Although *E. guineensis* and interspecific hybrid O×G cultivars exhibit a significant ability to recover from water stress, both species generally exhibit variable responses in terms of photosynthesis rate, gas exchange, stomatal conductance, and osmolyte concentration under water deficit conditions. These traits allow for the discrimination among oil palm cultivars capable of modulating their physiological and biochemical processes in response to drought stress [15,16,17]. The vast amount of information related to the physiological and biochemical responses of oil palm to water-limited conditions has been used for conventional breeding. However, the molecular mechanisms involved in drought stress tolerance in oil palm plants are not fully understood.

Recent studies based on marker-assisted selection and breeding strategies (genome-wide association studies [GWASs], quantitative trait locus mapping [QTL], and genome selection [GS]) have been used to obtain novel insights into oil palm drought stress responses. Hualkasin et al. [18] identified and cloned the *EgLEA4* gene and evaluated its expression in several oil palm tissues, suggesting that the EgLEA4 protein may be involved in the drought response pathway. Moreover, significant advances in identifying genetic mechanisms were achieved through GWASs associating genetic variants with a core of transcription factors (TFs), such as *WRKY* [19], *AP2/ERF* [20], *bZIP* [21], *ARF* [22], and *MYB* [23], expressed under drought stress conditions. Moreover, Wang et al. [24] explored the transcriptional response of oil palm roots to drought stress and identified 1293 genes, including 96 differentially expressed TFs. Furthermore, some oil palm gene targets have been introduced or edited in model plants to improve the response to limiting conditions. Recently, Leão et al. [25] applied multiomics integration (MOI) analyses to identify commonalities and differences in oil palm biomolecule responses related to drought and salt stress.

Integrated omics has been used to identify biomolecules associated with plant tolerance responses, such as clusters of sensors, receptors, phytohormones, genes, TFs, proteins, metabolites, and pathways [26]. Transcriptomics and gene coexpression networks are helpful and informative tools for analyzing differential gene expression. Exploring and identifying DEGs and pathways through computational and mathematical approaches might elucidate biological phenomena through temporal and spatial approaches [27,28]. Coexpression network analysis uses several measurements of centrality, degree, and modularity to infer biological functions and connections among genes; these interactions in the networks may represent regulator–target relationships or the functions of unknown genes or structural genes belonging to a specific metabolic pathway [29].

This work aimed to identify the genes, TFs, and pathways involved in the oil palm drought response using RNA-Seq and systems biology analysis and integrate them with physiological plant responses. We performed differential gene expression and genetic network analyses of two oil palm genotypes under water deficit conditions. In addition, a subset of 15 DEGs and network-relevant genes were selected for RT–qPCR validation. Our results provide a theoretical basis for understanding the molecular responses of oil palms to drought stress. These findings provide relevant information for the development of oil palm-tolerant cultivars, increasing the desired characteristics and preserving the genetic bases of oil palm.

## 2. Results

**Figure 1:**
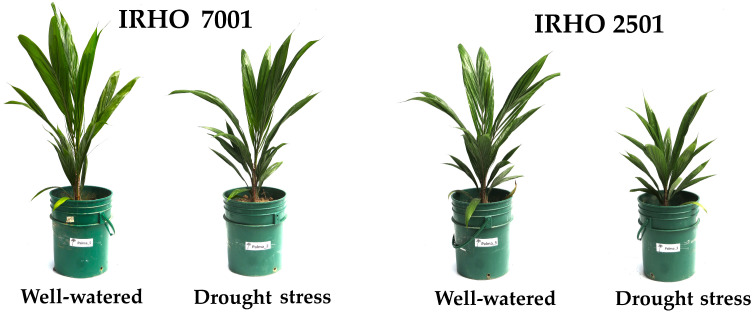
Physical appearance of two oil palm cultivars, Deli × La Mé, (IRHO 7001 and IRHO 2501) in response to water deficit. Ninety-day-old palms were maintained under field capacity (well-watered) or subjected to water deprivation for three weeks (drought stress).

**Figure 2:**
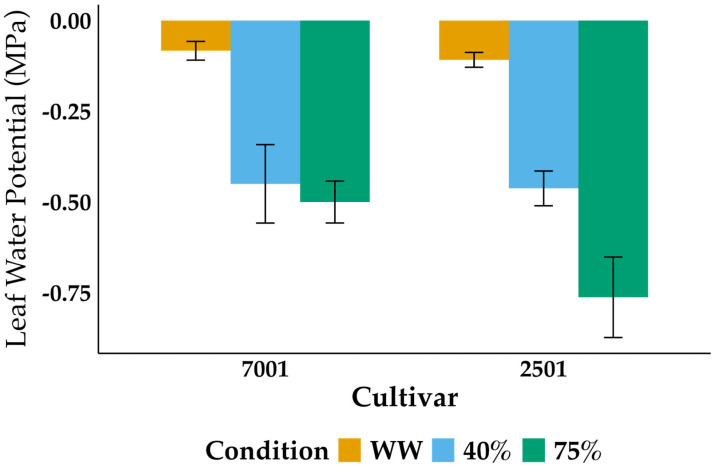
Predawn leaf water potential (Ψleaf) of two oil palm cultivars, Deli × La Mé (IRHO 7001 and IRHO 2501), in response to water deficit. Ninety-day-old palms were maintained under field capacity (well-watered) or subjected to water deprivation until the photosynthetic rate of the IRHO 7001 cultivar dropped 40% (40%), which is considered moderate drought stress, or until it dropped 75% (75%), which is considered severe drought stress. Each box corresponds to the mean ± SD (*n* = 6).

Water deficit induced changes in the growth of oil palm. Based on the physical appearance of the plants, cultivar IRHO 2501 was more affected than IRHO 7001 (Figure 1). The changes in leaf water potential (Ψleaf) further corroborated the cultivars’ differential response (Figure 2). Thus, under well-watered conditions, the Ψleaf of both cultivars was similar, with values of −0.0833 ± 0.0258 MPa for IRHO 7001 and −0.108 ± 0.0204 MPa for IRHO 2501. In response to drought stress conditions, the cultivars reduced their Ψleaf. Under moderate drought stress, the reduction was similar in both cultivars, with values of −0.45 ± 0.108 MPa for IRHO 7001 and −0.462 ± 0.0479 MPa for IRHO 2501. However, when the drought stress progressed to severe, the Ψleaf in IRHO 7001 showed a moderate drop to −0.5 ± 0.0577 MPa, while the reduction in IRHO 2501 was a more pronounced drop to −0.762 ± 0.111 MPa. In this case, highly significant differences existed among cultivars, the stress condition, and the interaction (Table 1).

### 2.1. Physiological Traits

**Figure 3:**
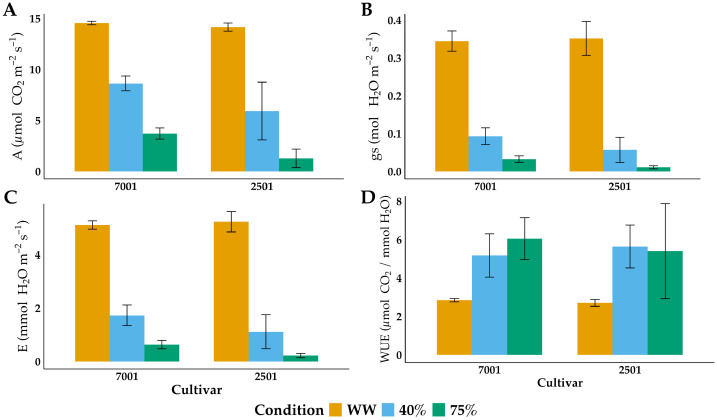
Physiological response of two oil palm cultivars, Deli × La Mé, IRHO 7001 (7001) and IRHO 2501 (2501) in response to water deficit. Ninety-day-old palms were maintained under field capacity (well-watered) or subjected to water deprivation until the photosynthetic rate of the IRHO 7001 cultivar dropped 40% (40%), which is considered moderate drought stress, or until it dropped 75% (75%), which is considered severe drought stress. Each box corresponds to the mean ± SD. (*n* = 6). (**A**). photosynthetic rate (*A*), (**B**). stomatal conductance (*gs*), (**C**). transpiration rate (E), and (**D**). instantaneous leaf-level water use efficiency (WUE).

The physiological parameters of both cultivars were quite similar under WW conditions, indicating an optimum water content in the plants (Figure 3). On average, the baseline photosynthetic rate was close to 15 μmol CO_2_ m^−2^ s^−1^ (Figure 3A), the stomatal conductance was close to 0.35 mol H_2_O m^−2^ s^−1^ (Figure 3B), the transpiration rate was slightly greater than 5.0 mmol H_2_O m^−2^ s^−1^ (Figure 3C), and the instantaneous leaf-level water use efficiency was close to 2.8 mol CO_2_ mol^−1^ H_2_O (Figure 3D).

The cultivars’ physiological responses were different under water deficit conditions. Thus, when the photosynthetic rate in IRHO 7001 was reduced by 40%, the reduction in IRHO 2501 was 58% (moderate drought stress). When *A* was decreased by 75% in IRHO 7001, the reduction in IRHO 2501 was 91% (severe drought stress) (Figure 3A).

In both cultivars, *gs* presented similar behavior under drought stress treatments, and no differences were found between IRHO 7001 and IRHO 2501 (*p* ≥ 0.2183). However, significant differences were found in *gs* under the stress treatments. When the photosynthetic rate decreased by 40%, *gs* decreased by approximately 72% and 85% in response to WW in IRHO 7001 and IRHO 2501, respectively. Moreover, in the severe drought stress treatment, *gs* decreased by 90% in IRHO 7001 and 97% in IRHO 2501 (Figure 3B)

Concerning the transpiration rate (*E*), we observed a significant reduction in the transpiration rate when the photosynthetic rate decreased by 40% (*p* ≤ 0.001). In IRHO 7001, the reduction in *E* was close to 65% and 87% under moderate and severe drought stress, respectively, while in IRHO 2501, the decrease in *E* was 78% and 96%, respectively (Figure 3C). Regarding the instantaneous leaf-level WUE, the tolerant cultivar IRHO 7001 used water more efficiently than the sensitive cultivar IRHO 2501 under WW conditions (Figure 3D).

### 2.2. Library Construction and Differential Gene Expression

**Figure 4:**
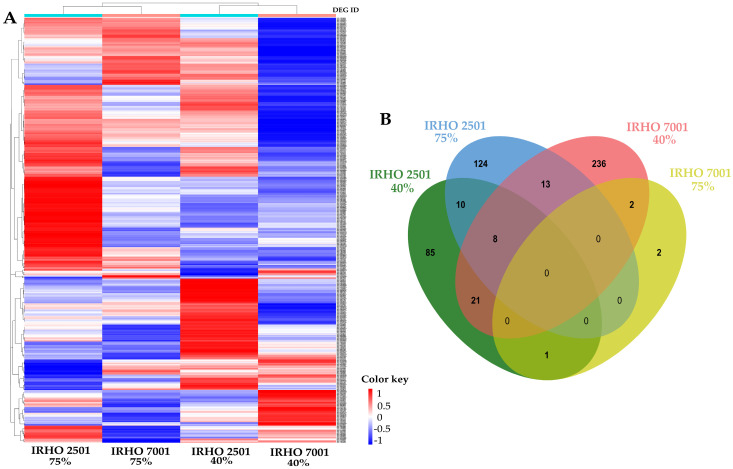
DEGs of two oil palm cultivars, Deli × La Mé, (IRHO 7001 and IRHO 2501) in response to water deficit. Ninety-day-old palms were maintained under field capacity (well-watered) or subjected to water deprivation until the photosynthetic rate of the IRHO 7001 cultivar dropped 40% (40%), which is considered moderate drought stress, or until it dropped 75% (75%), which is considered severe drought stress. (**A**) Heatmap of the RNA-Seq samples. A tendency toward red indicates under-expression, while a tendency toward blue indicates overexpression. (**B**) Unique and shared DEGs between two contrasting oil palm genotypes and drought stress conditions. The color key scale corresponds to the L2FC, tendency to blue correspond to underexpressed genes, while tendency to red indicates overexpressed.

The sequencing generated 151 pb paired end reads. RNA-Seq generated 144 Gb of raw information from 24 samples (Supplementary Table S1). The count range was between 122,172,034 and 209,146,404 paired reads per sample. The DESeq2 algorithm identified 502 DEGs (*p* value ≤ 0.1 and LFC |2|) between IRHO 7001 and IRHO 2501 under moderate and severe drought stress (Supplementary Table S2). According to the heatmap, the expression profiles differed between IRHO 7001 and IRHO 2501. Remarkably, an extensive list of genes related to IRHO 2501 was found to be overexpressed in IRHO 7001 under moderate drought stress conditions (Figure 4A). Under moderate drought stress conditions, 236 unique genes induced by moderate drought stress conditions were identified in the IRHO 7001 cultivar. According to Venn diagram analysis, 85 unique genes were identified in the IRHO 2501 cultivar (Figure 4B). Under moderate drought stress conditions, 21 genes were shared between the IRHO 7001 and IRHO 2501 cultivars. Interestingly, in the IRHO 7001 cultivar, only two genes were shared between moderate and severe drought conditions, suggesting different response mechanisms according to drought stress conditions.

### 2.3. Gene Coexpression Networks

**Figure 5:**
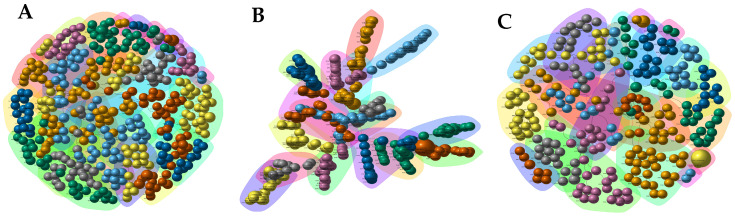
Gene coexpression networks of two oil palm cultivars, Deli × La Mé, (IRHO 7001 and IRHO 2501) in response to water deficit. (**A**) General; (**B**) IRHO 7001; and (**C**) IRHO 2501. The igraph R package was used to construct the general and specific cultivar coexpression networks under drought stress. Each node (sphere or bead-like shape) represents a gene, and groups of nodes highlighted with the same color indicate a module of genes. The black edges represent direct correlations between genes, and the red lines represent inverse correlations. The size of each node is proportional to the mean expression level of the gene represented by the node.

The constructed gene coexpression networks included all 502 DEGs. The first network corresponded to the general gene coexpression network, where we included all DEGs from both cultivars; the second coexpression network included only DEGs from the IRHO 7001 cultivar; and the third network included only DEGs from the IRHO 2501 cultivar (Supplementary Table S3). A general gene coexpression network was constructed with 374 DEGs (Figure 5A). This network predicted 28 modules and six overexpressed HUB genes, among which the zinc finger protein ZAT11 had the highest HUB score. The specific gene coexpression network for the IRHO 7001 cultivar was constructed with 274 DEGs (Figure 5B). This network predicted 16 modules and 46 HUB genes, among which serine threonine-protein phosphatase 6 regulatory ankyrin repeat subunit B was the most representative gene in the HUB score. In contrast, the specific gene coexpression network for the IRHO 2501 cultivar was built with 249 DEGs, 20 predicted modules, and three HUB genes (Figure 5C). In this network, the DUF1677 family protein had the highest HUB score.

### 2.4. Validation of DEGs by Quantitative Real-Time PCR

**Figure 6:**
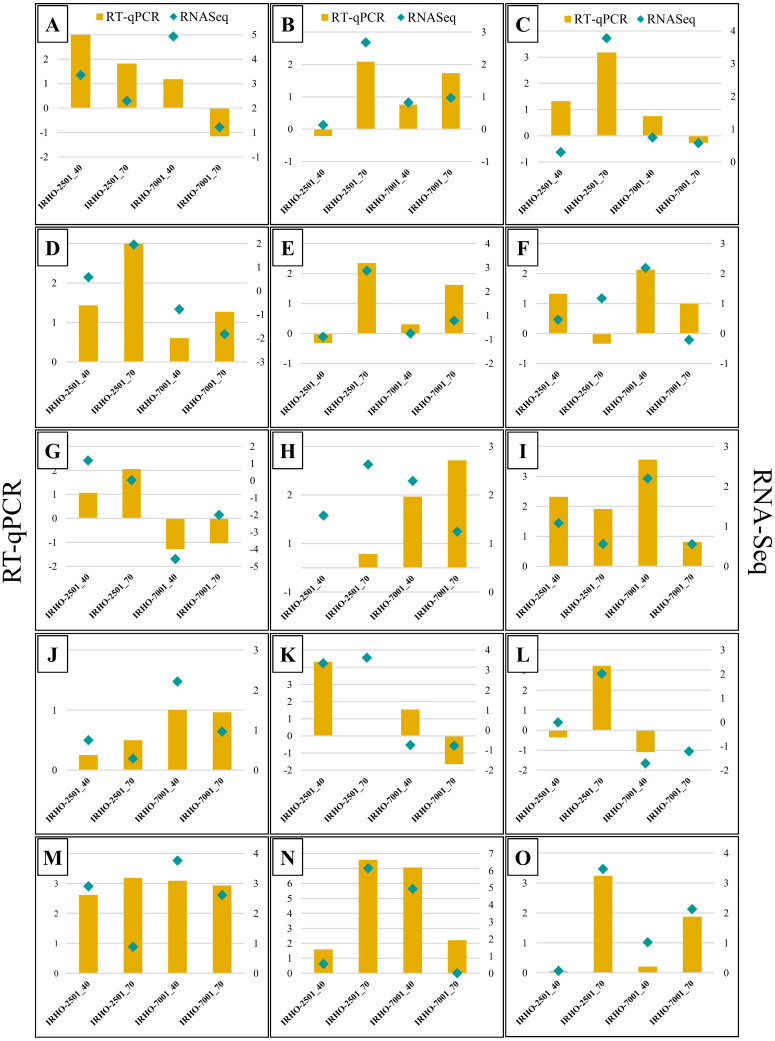
Relative quantification of 15 genes by RT–qPCR compared against RNA-Seq in two oil palm cultivars, Deli × La Mé, (IRHO 7001 and IRHO 2501) in response to water deficit. Ninety-day-old palms were maintained under field capacity (well-watered) or subjected to water deprivation until the photosynthetic rate of the IRHO 7001 cultivar dropped 40% (40%), which is considered moderate drought stress, or until it dropped 75% (75%), which is considered severe drought stress. Yellow bars indicate the relative expression value obtained by RT-qPCR. Lite blue diamonds indicate the RNA-Seq value. (**A**) WRKY transcription factor 51; (**B**) NAC transcription factor NAM-B2-like_ NAM-B2; (**C**) beta-xylosidase alpha-L-arabinofuranosidase 2-like OsI_08964_ BXL1; (**D**) Leucine-rich repeat receptor-like serine_ At1g17230; (**E**) Calcium-binding protein CML42; (**F**) Ser/threo-protein phosphatase 6 regulatory ankyrin repeat subunit B; (**G**) Pectinesterase-like; (**H**) Pentatricopeptide repeat-containing protein_ At5g39980; (**I**) Multiple C2 and transmembrane domain-containing protein 2-like, (**J**) Non-specific lipid-transfer protein 2-like; (**K**) Transcription factor bHLH35-like isoform X1; (**L**) Mitogen-activated protein kinase kinase kinase 2-like; (**M**) Bidirectional sugar transporter SWEET14-like; (**N**) Galactinol synthase 1-like_ GOLS1; and (**O**) Xyloglucan endotransglucosylase/hydrolase protein 22-like XTH22.

We evaluated the expression of 15 DEGs by RT–qPCR to verify the RNA-Seq data (Figure 6; Supplementary Figure S1). A strong association (r = 0.87) was found between the RNA-Seq and RT–qPCR data. We did not observe significant differences in the expression of the housekeeping genes GAPDH or EF1 between the treatments or genotypes (Kruskal–Wallis test *p*-value = 0.241 and 0.131, respectively). This indicates that the expression of GAPDH and EF1 was stable between genotypes and treatments, and these genes were used as reference genes. This result suggests that the transcriptomic data are reliable.

## 3. Discussion

Drought stress due to climate change is a serious threat to the sustainable cultivation of oil palm. To overcome the adverse effects caused by water deficit, oil palm cultivars exhibit several physiological, biochemical, and molecular mechanisms that help them avoid, tolerate, or escape from limiting conditions. The genetic improvement in oil palm drought-tolerant cultivars might mitigate climate change impacts on cultivation and optimize oil palm yield. Here, we investigated the effects of moderate and severe drought stress conditions on two commercial oil palm cultivars in the nursery. The changes observed in the phenotypic and physiological parameters, complemented by the transcriptome and gene coexpression network analysis of the commercial IRHO 7001 and IRHO 2501 cultivars, provided new insights into drought stress tolerance in oil palm.

The cultivars’ differential physiological responses to drought stress demonstrate the tolerant and susceptible characteristics of IRHO 7001 and IRHO 2501, respectively. The smaller reduction in leaf water potential in IRHO 7001 compared with IRHO 2501 under severe drought stress indicates tolerance to water stress conditions [11,15,30]. This was corroborated by the higher reduction in the photosynthetic rate in IRHO 2501 under moderate and severe drought stress compared with IRHO 7001. However, the fact that the other gas exchange parameters, stomatal conductance, transpiration, and water use efficiency of photosynthesis were not statistically different between the cultivars implies biochemical and molecular differences between the two cultivars [11], which are responsible for the tolerance or susceptibility observed.

The transcriptomics profiles and gene coexpression network approaches predicted a set of DEGs, and the HUB genes associated with regulatory and transcriptional functions. The most important genes in the general coexpression network were the zinc finger protein *ZAT11* and linoleate 13S-lipoxygenase 2-1 2C *LOX2.1*. These genes were found to be overexpressed in IRHO 2501 but under-expressed in IRHO 7001. The ZAT11 protein, a type of C2H2-type zinc finger, is a member of a family of specific transcriptional regulator genes with dual functions associated with growth, development, and stress resistance in cotton [31]. The plant lipoxygenase (LOX) family has been associated with several physiological processes, such as growth, pest resistance, and senescence, through the catalysis of the hydroperoxidation of lipids [32]. In this order, the overexpression of both *ZAT11* and *LOX2.1* possibly corresponds to an antagonistic mechanism response, contributing to the sensitive response of the IRHO 2501 cultivar to drought stress and becoming an interesting target for genome editing approaches.

The gene coexpression network for the IRHO 2501 cultivar showed the *DUF1677* family proteins and serine and threonine-protein kinase-like protein *CCR4* as the most significantly upregulated genes. The *DUF1677* gene belongs to a family of hypothetical proteins expressed in transgenic rice and *Arabidopsis* as a tolerance response to drought stress [33]. *CCR4* is a protein kinase involved in signal transduction, regulation, and senescence [34,35]. The *DUF1677* family and *CCR4* in oil palm have yet to be studied, and their functions still need to be elucidated. In the IRHO 7001 gene coexpression network, the genes encoding serine threonine-protein phosphatase 6 regulatory ankyrin repeat subunit B and beta-galactosidase-like 2C were predicted to be the most important genes for this mathematical modeling. The phosphatase holoenzyme serine–threonine protein phosphatase 6 (PP6) is an important reversible post-translational subunit involved in the photoperiodic control of flowering and directional transport of the phytohormone auxin in *Arabidopsis*, as reviewed by Bheri et al. [36]. However, additional research is required to determine the function of the ankyrin repeat domain function of PP6 in oil palm in association with the abiotic stress response. On the other hand, beta-galactosidase-like 2C (*BGAL*) was found to be under-expressed; this gene is a glycosyl hydrolase involved in cell wall reorganization and fruit ripening and softening induced by ethylene and methyl jasmonate (MeJA) in apples [37]. Under these stress conditions, this gene might be downregulated by other phytohormone signaling processes.

Interestingly, the bidirectional sugar transporters *SWEET14-like* and galactinol synthase 1-like *GolS1* were overexpressed under moderate and severe drought stress in IRHO 7001 and IRHO 2501, respectively. In addition, in the specific gene coexpression network of IRHO 7001, these genes were predicted to be HUB genes with the highest HUB score for their modules, suggesting that these genes have essential functions in the response to drought stress in oil palm. The SWEET gene family comprises monosaccharide and sucrose transporter proteins that help plants balance cell osmotic potential and regulate abiotic stress responses by transporting and redistributing soluble sugars [38]. On the other hand, the *GOLS1* gene encodes an important enzyme involved in raffinose family oligosaccharide (RFO) biosynthesis. Among RFOs, raffinose has been directly and positively associated with abiotic stress tolerance under long-term natural conditions [39]. In this sense, preserving cell osmotic potential through the biosynthesis and redistribution of sugars and proline in oil palm is a common response mechanism [11,14]. The molecular basis of the significant ability of oil palm plants to recover from water stress might involve the overexpression of *SWEET*, *GOLS1*, and amino acid transporters.

Water deficiency in soils is primarily sensed by roots, which play a significant role in the systemic response and mediating root–shoot communication [40]. Once plant roots detect an initial drought signal, signal transduction is induced by a wide range of long-distance signal traducers, including nutrients (calcium Ca^2+^), reactive oxygen species (ROS), nucleic acids (miRNAs), proteins, or phytohormones [41]. Under drought stress conditions, the biosynthesis and accumulation of phytohormones are significantly disrupted. However, the specific production and combinations of several phytohormones, such as abscisic acid (ABA), ethylene (ET), auxin (AUX), cytokinins (CKs), salicylic acid (SA), jasmonic acid (JA), gibberellic acid (GA), brassinosteroids (BRs), and strigolactones (SLs), cause the detrimental effects of abiotic stress and enhance the stress tolerance response [42].

Plasma membrane receptors may perceive drought stress conditions in oil palm plants and trigger a signaling process from roots to leaves mediated by phytohormones or ROS. Stomatal closure is the key symptom of water deficit and is highly sensitive to fluctuating air humidity, temperature, and several signaling substances [39]. Here, the abscisic acid receptor PYL4-like *PYL4*, involved in stomatal closure, was found to be under-expressed, suggesting the activity of other phytohormone mechanisms. The genes encoding the membrane receptors leucine-rich repeat receptor-like protein kinase *PXC1* and histidine kinase 4-like *HK4* were found to be overexpressed only in the tolerant cultivar IRHO 7001, indicating that these genes are important for signaling reception and subsequent drought stress tolerance. The *PXC1* gene triggers the ROS- or ABA-dependent pathway and regulates nutrient flux into guard cells to promote stomatal closure. Simultaneously, *PXC1* and *HK4* might trigger kinase/phosphatase protein signaling and activate or deactivate response pathways associated with several phytohormones.

**Figure 7:**
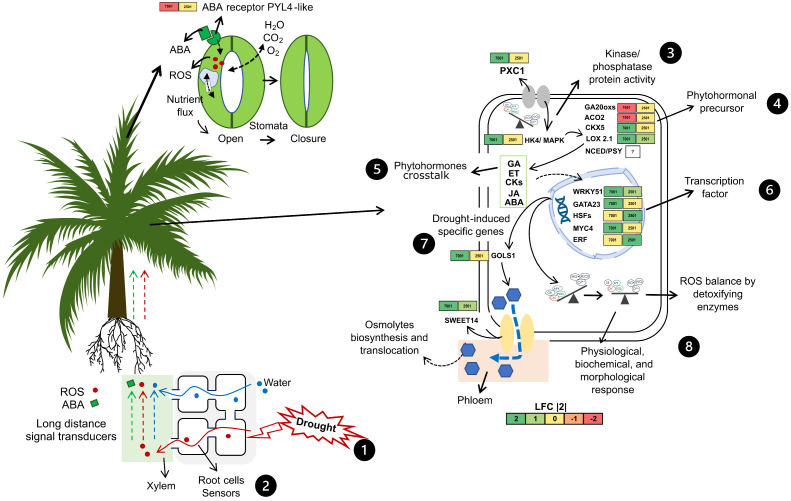
Phytohormone crosstalk and signal cascades of two oil palm cultivars, Deli × La Mé, (IRHO 7001 and IRHO 2501) in response to water deficit. The numbers indicate the step-by-step signaling cascade response in oil palms under drought stress. Numbers 1 and 2 indicate the stimulus and signal perception. 3 indicates signal transduction. 4, 5, and 6 indicate phytohormone metabolism and TFs activation/ inactivation. 7 and 8 indicate drought stress-induced genes and ROS metabolism balance. Gene expression levels are indicated for each cultivar, where 7001 = IRHO 7001 and 2501 = IRHO 2501. The square color corresponds to the gene expression color scale in the L2FC bar. The question mark indicates no gene expression. Arrows colors indicate flux of water (blue), ABA (green), and ROS (red) from soil or roots to leaves. The figure was partly generated using plant icon adaptations licensed and created by Guillaume Lobet (https://figshare.com/authors/Plant_Illustrations/3773596 is licensed under CC-BY 4.0 Unported https://creativecommons.org/licenses/by/4.0/, accessed on 16 April 2024).

In oil palm, the drought signaling cascade and phytohormone crosstalk among GA, ET, CK, JA, and ABA play central roles in oil palm response and adaptation to drought stress conditions. It is possible that HK4 negatively regulates phytohormone precursors such as the gibberellin 20 oxidase 1-D-like gene *GA20ox1* and the 1-aminocyclopropane-1-carboxylate oxidase *ACO2*, which are involved in gibberellin and ethylene biosynthesis, respectively. On the other hand, HK4 could induce the overexpression of genes related to phytohormone precursors such as cytokinin dehydrogenase 5-like *CKX5* and linoleate 13S-lipoxygenase 2-1 *LOX2.1*, which are involved in CK and JA biosynthesis. In this sense, the biosynthesis of CKs and JA and the negative regulation of GA, ET, and ABA might induce several TF’ under-expression or overexpression. Here, the putative WRKY transcription factor 51 *WRKY51*, the transcription factor MYC4-like *MYC4*, the GATA transcription factor 23-like *GATA23*, the heat stress transcription factor A-2b *HSFA2B*, and the two-component response regulator ARR12-like *ARR12* were found to be overexpressed in the tolerant cultivar IRHO 7001, suggesting a tolerance response (Figure 7).

WRKY TFs interact with other TFs to mediate several stress conditions and development. *WRKY51* is an ABA-inducible and GA-repressible gene that regulates the crosstalk between GA and ABA signaling in embryos by repressing RAmy1A α-amylase [43]. The *MYC4* regulates *bHLH* TFs and interacts with *MYC2* and *MYC3* to activate JA responses [44]. Interestingly, the overexpression of *GATA23* is involved in lateral root development induced by auxin regulatory components; perhaps *GATA23* possesses a paralogous function and is activated in leaves [45]. Overall, the overexpression or under expression of all these TFs might contribute directly or indirectly to the expression regulation of drought-induced genes.

Finally, we found a possible drought stress response mediated by lipid metabolism. Acyl-lipids are components of cellular membranes that help plants sense extracellular conditions, playing a crucial role in the stimulus response of plant cells. The possible roles of glycerol-3-phosphate acyltransferase 5-like *GPAT5* and the glycerophosphodiester phosphodiesterase *GDPD1* in cellular lipid signaling may contribute to overcoming drought stress conditions. The genes *GPAT5* and *GDPD1* were found to be overexpressed only in the tolerant cultivar IRHO 7001. In *Arabidopsis*, the overexpression of SsGPAT5 alleviates the photoinhibition of PSII and PSI under salt stress, while the GDPD family has been associated with cellular phosphate homeostasis [46,47]. Further research is required to reveal the role of lipids as signaling molecules or drought mitigators during the stress response in plants.

In summary, the analysis of the transcriptome and gene coexpression networks allowed us to identify genes involved in the response of oil palm to water deficit stress. In oil palm, the synchronized regulation of several TF and phytohormone precursor genes directs the drought stress signaling cascade. Phytohormone crosstalk plays a central role in the response and adaptation of oil palm plants to water stress conditions. These findings provide a basis for functionally characterizing candidate genes associated with drought stress tolerance in oil palm. However, a better understanding of the oil palm response and new breeding techniques are crucial for generating drought stress-tolerant oil palm cultivars and improving productivity under limiting conditions.

## 4. Materials and Methods

### 4.1. Plant Material and Experimental Conditions

This study was conducted in a mesh house measuring 20 × 10 × 3 m, featuring a polycarbonate roof and five mesh walls. Within the mesh house, the relative humidity dropped below 50%, and temperatures rose to 35 °C. To maintain a minimum relative humidity of 50% and ensure optimal conditions for photosynthesis, external humidifiers were installed, which also helped to keep the temperature below 32 °C.

Two commercial *E. guineensis* Deli × La Mé cultivars, IRHO 7001 and IRHO 2501, were evaluated under two drought stress conditions. Pregerminated seeds with differentiated plumules and radicles (at the 004 phenological stage) were kept in germination soil until the seedlings developed five lanceolate leaves (at the 109 phenological stage), according to the BBCH scale for African oil palm [48]. Then, the seedlings were transplanted into plastic containers with 20 kg of soil composed of 42.1% sand, 21.4% silt, and 36.4% clay and a 1.22 g/cm^3^ soil density. Seedlings were maintained under field capacity conditions for 90 days to adapt to the new conditions. Then, the plants were kept under field capacity conditions (well-watered, WW) or deprived of water (water deficit conditions). The plants’ photosynthetic rate (*A*) was monitored daily using a portable photosynthesis meter LI-6800 open-path portable photosynthesis system (LiCor, Inc., Lincoln, NE, USA). Two sample times were defined based on photosynthetic rate decline in the plants subjected to water deficit. One sample time was when *A* dropped 40% (one week after water deprivation onset), and the second was when A dropped 75% (three weeks after water deprivation onset) in the cultivar IRHO 7001, previously described as water stress-tolerant [11]. Sample time one was moderate drought stress, and sample time two was severe drought stress. The well-watered plants were considered controls.

### 4.2. Physiological Measurements

The LI-6800 open-path portable photosynthesis system (LiCor, Inc., Lincoln, NE, USA) was used to record the net assimilation rate of CO_2_ or the photosynthetic rate (*A*), stomatal conductance (*gs*), and transpiration rate (*E*). The following parameters were fixed at the measuring points: 28 °C and 65% of the temperature and relative humidity of the chamber, an airflow rate of 300 μmol s^−1^, a CO_2_ concentration in the chamber of 400 ppm, and a PAR of 1000 μmol photons m^−2^ s^−1^. The measurements were taken on the third leaf of the palms in the morning between 9:00 and 11:00. The instantaneous leaf-level water use efficiency (WUE) was determined by the ratio of *A* to *E* (*A*/*E*). The leaf water potential (Ψleaf) was determined using a Model 3005 Plant Water Status Console device (Soilmoisture Equipment, Santa Barbara, CA, USA) between 4:00 and 6:00 h (predawn).

### 4.3. RNA Isolation and Library Construction

Immediately after physiological measurements, leaf samples were collected and homogenized with liquid nitrogen for RNA isolation. Total RNA was extracted from 100 mg of frozen leaf tissue using the NucleoSpin RNA Plant and Fungi Mini Kit for RNA from plants and fungi (Macherey-Nagel SAS, Dueren, Germany) according to the manufacturer’s protocol. Total RNA concentration and quality were examined using an Agilent 2100 Bioanalyzer (Santa Clara, CA, USA). RNA samples with an RIN ≥ 6 were precipitated with 3 M sodium acetate and ethanol and processed by Macrogen (Seoul, Republic of Korea) for RNA-Seq library construction. The libraries were constructed using a TruSeq Stranded mRNA LT (Illumina, Inc., San Diego, CA, USA) and sequenced on the Illumina NovaSeq 6000 platform (Illumina, Inc., San Diego, CA, USA).

### 4.4. Differential Gene Expression and Gene Coexpression Network Construction

Raw transcriptomic FASTQ files of 24 leaf samples were analyzed with the DESeq2 algorithm in the R package [49]. To identify DEGs, *p*-adj ≤ 0.1 and Log2FoldChange ≥ |2| were set as thresholds for significant DEGs according to the DESeq2 algorithm. Sequences were aligned against an in-house oil palm genome with TopHat2 software (V2.1.1) [50] using the “SummarizeOverlaps” function to count aligned overlapping sequences against the oil palm genome reference. Heatmaps and Venn diagrams were used to group samples and identify unique and shared genes between cultivars.

The igraph R package was used to construct the global and cultivar-specific coexpression networks. Each network was built based on the correlation of all gene pair distances among the normalized read counts, and only DEGs were used. An edge among genes was considered when the correlation was ≥0.8. The “edge betweenness” function identified communities or modules of genes correlated among them [51]. HUB genes were identified according to an HUB score ≥ 0.1 using the algorithm developed by Kleinberg [52]. Additional metrics of the networks were estimated by density, diameter [53], diameter (weighted), centralization degree, centralization closeness, centralization betweenness [54,55], and average path length [56].

### 4.5. Validation of DEGs by Quantitative Real-Time PCR

RNA-Seq transcriptome analysis confirmed 15 selected genes by RT–qPCR. The genes were selected based on the following parameters: (1) high HUB score in the gene coexpression network, (2) contrasting expression among genotypes, and (3) previous reports in the literature indicating that HUB is important for drought stress tolerance in related plants (target and housekeeping gene sequences are listed in Supplementary Table S4). Primer3web V4.1.0 (https://primer3.ut.ee/, accessed on 28 July 2023) [57,58,59] was used to design primer sequences following the Minimum Information for Publication of Quantitative real-time PCR Experiments (MIQE) guidelines [60]. After 1 µg of RNA was treated with DNase I (Invitrogen™, Waltham, MA, USA), cDNA synthesis was performed using SuperScript™ IV Reverse Transcriptase (Invitrogen™, Waltham, MA, USA) following the manufacturer’s recommendations. Genes with efficiencies greater than 85% and one defined melting peak were validated. qPCR was performed in 10 µL of reaction mixture using Fast Evagreen^®^ qPCR Master Mix (Biotium, Inc., Fremont, CA, USA) in a real-time QIAquant 96 5plex (Qiagen, Germantown, MD, USA) following the manufacturer’s recommendations. The relative expression of each gene was calculated using the delta–delta Ct method (2^∆∆Ct^), and the GAPDH and EF1 genes were used as normalizers. The association between the RNA-Seq and RT–qPCR results was established by the correlation coefficient (r).

### 4.6. Statistical Analysis

The experiment was conducted under a completely randomized split-plot design with continuous sampling. The whole plots were the cultivars (two levels, IRHO 7001 and IRHO 2501). The assigned subplots were the drought conditions (well-watered, 40%, 70%), corresponding to moderate drought stress, defined as the time when water deprivation induced a 40% drop in A in the cultivar IRHO 7001, and severe drought stress when water deprivation induced a 70% drop in A in the same cultivar. The experimental unit was composed of six plants per treatment. We performed an analysis of variance (ANOVA) using the general linear model (GLM) procedure in SAS software Version 9.4M8 to test differences in physiological and biochemical responses between stress conditions and cultivars. Mean comparisons were made through Tukey’s Studentized Range (HSD) test. A nonparametric Kruskal–Wallis test (*p* < 0.05) was performed to compare whether the mean GAPDH and EF1 expression Ct values were stable between the stress conditions and cultivars via R software Version 4.3.0.

## Figures and Tables

**Table 1:** Summary of ANOVA results for the physiological variables. Ψleaf: leaf water potential, A: photosynthetic rate, *gs*: stomatal conductance, E: transpiration rate, WUE: instantaneous leaf-level water-use efficiency. Significance levels. ns: not significant; ** significantly different *p* ≤ 0.01; *** highly significantly different *p* ≤ 0.001.

Physiological Parameter	Source	df	F-Value	*p*-Value	Significance
Ψ_leaf_	Cultivar	1	13.037	0.00155	**
	Condition	2	175.016	3.09 × 10^−14^	***
	Cultivar × condition	2	9.835	0.00089	***
A	Cultivar	1	13.868	0.00118	**
	Condition	2	260.053	4.92 × 10^−16^	***
	Cultivar × condition	2	2.988	0.07112	ns
*gs*	Cultivar	1	1.454	0.24100	ns
	Condition	2	367.480	2.00 × 10^−16^	***
	Cultivar × condition	2	1.415	0.26400	ns
E	Cultivar	1	3.329	0.08170	ns
	Condition	2	534.337	1.99 × 10^−16^	***
	Cultivar × condition	2	3.074	0.06650	ns
WUE	Cultivar	1	0.066	0.80000	ns
	Condition	2	19.957	1.14 × 10^−5^	***
	Cultivar × condition	2	0.466	0.63300	ns

## Data Availability

The datasets generated and analyzed during the current study are available in the SRA under Bioproject PRJNA1137935.

## Supplementary Materials

The following supporting information can be downloaded at https://www.mdpi.com/article/10.3390/ijms25168761/s1.
