# Supplementary material for: Integrative Analysis of Transcriptomic Profiles and Physiological Responses Provide New Insights into Drought Stress Tolerance in Oil Palm (Elaeis guineensis Jacq.)
Source: Int J Mol Sci. 2024 Aug 12;25(16):8761. doi: 10.3390/ijms25168761 (PMC11354634; doi:10.3390/ijms25168761)
Supplement: Supplementary file 1 [file ijms-25-08761-s001.zip › Tables and Figure caption.pdf]

Association between the RNA-Seq and RT–qPCR data. (Supplementary Figure S1)

Raw information of RNA-Seq from 24 samples (Supplementary Table S1)

Differentially expressed genes identified between IRHO 7001 and IRHO 2501 under moderate and severe drought stress by The DESeq2 algorithm (Supplementary Table S2).

All differentially expressed genes in constructing gene coexpression networks (Supplementary Table S3).

Target and housekeeping gene sequences (Supplementary Table S4).
