# Supplementary figures and images for: Integrative Analysis of Transcriptomic Profiles and Physiological Responses Provide New Insights into Drought Stress Tolerance in Oil Palm (Elaeis guineensis Jacq.)
Source: Int J Mol Sci. 2024 Aug 12;25(16):8761. doi: 10.3390/ijms25168761 (PMC11354634; doi:10.3390/ijms25168761)

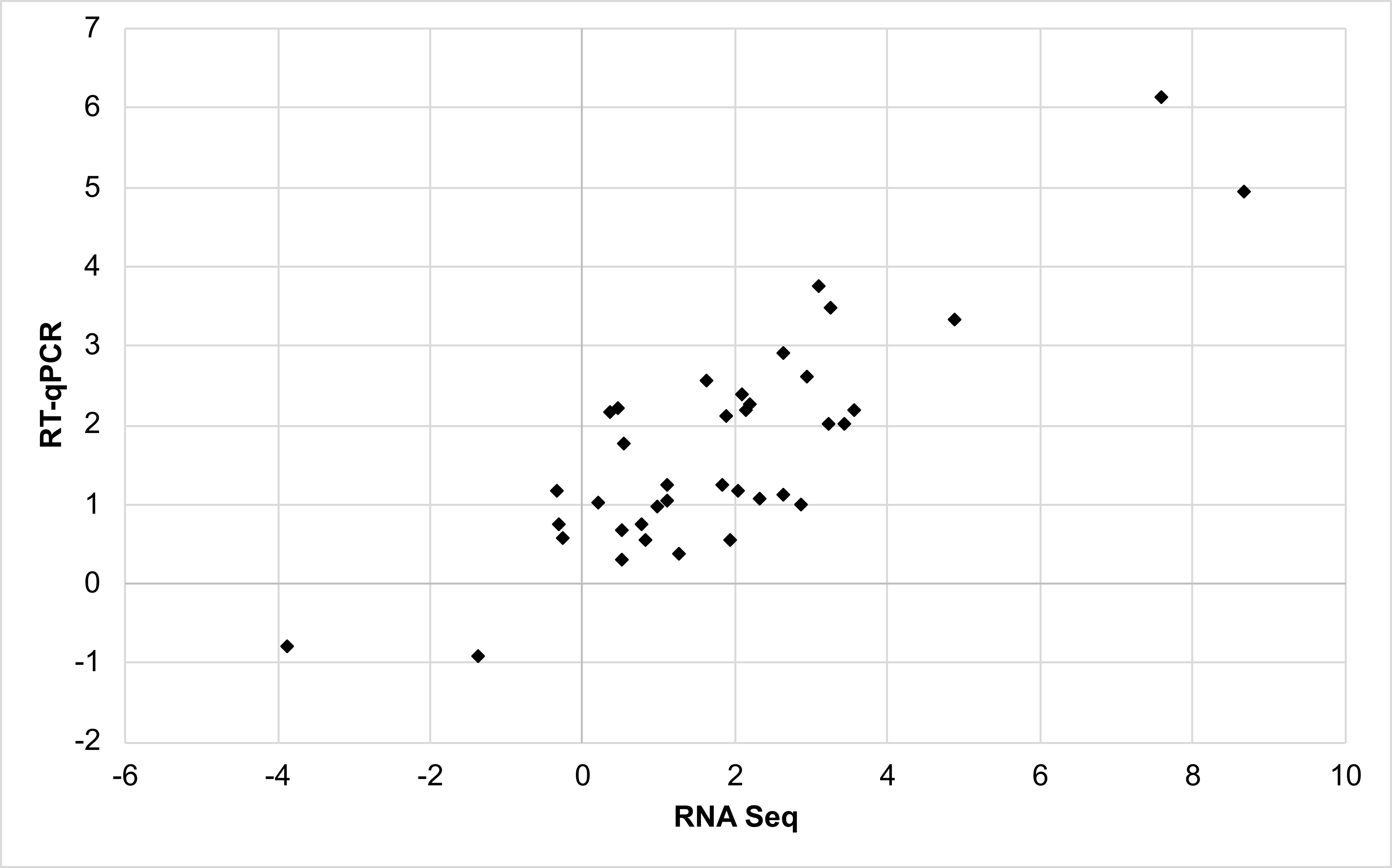

Supplement: Supplementary file 1 [file ijms-25-08761-s001.zip › Figure S1.tif]
